# Advances in Single-Cell Transcriptomics for Livestock Health

**DOI:** 10.3390/vetsci13020161

**Published:** 2026-02-06

**Authors:** Muhammad Zahoor Khan, Mohamed Tharwat, Abd Ullah, Fuad M. Alzahrani, Khalid J. Alzahrani, Khalaf F. Alsharif, Fahad A. Alshanbari

**Affiliations:** 1 College of Agriculture and Biology, Liaocheng University, Liaocheng 252000, China; 2 Department of Clinical Sciences, College of Veterinary Medicine, Qassim University, Buraidah 51452, Saudi Arabia; atieh@qu.edu.sa; 3 Department of Clinical Laboratories Sciences, College of Applied Medical Sciences, Taif University, Taif 21944, Saudi Arabia; 4 Department of Medical Biosciences, College of Veterinary Medicine, Qassim University, Buraidah 51452, Saudi Arabia

**Keywords:** cell atlas, host pathogen interaction, livestock immunity, single-cell RNA sequencing, vaccine development

## Abstract

RNA sequencing (scRNA-seq) has emerged as a transformative technology for dissecting cellular heterogeneity and immune complexity in livestock species. This review summarizes recent advances in the application of single-cell transcriptomics to livestock health, with a particular focus on immune system organization and host–pathogen interactions in cattle, pigs, poultry, and small ruminants. We highlight the development of large-scale, multi-tissue cell atlases—such as the Cattle Cell Atlas and resources generated through the Farm Animal Genotype-Tissue Expression (FarmGTEx) consortium—that provide foundational reference frameworks for livestock genomics. These atlases have enabled the identification of tissue- and species-specific immune cell populations, clarified cellular tropism of major bacterial and viral pathogens, and revealed distinctive immunological features, including the prominent role of γδ T cells in ruminant immunity. We discuss how single-cell immune receptor sequencing has advanced monoclonal antibody discovery and informed rational vaccine design. Key technical and analytical challenges, including incomplete genome annotations, tissue processing constraints, and cross-platform data integration, are critically assessed. Finally, we outline future directions integrating spatial transcriptomics and multi-omics approaches to further resolve immune function within tissue contexts. Collectively, these advances position single-cell transcriptomics as a central framework for improving disease resistance, vaccine efficacy, and translational research in livestock health.

## Simple Summary

Animals such as cattle, pigs, sheep, and goats are essential for global food security, but infectious diseases threaten their health and productivity. Understanding how these animals fight infections has been difficult because traditional laboratory methods cannot distinguish between the many different types of immune cells working together to protect the body. A new technology allows scientists to examine thousands of individual cells one at a time, revealing the complete diversity of immune cell types and how each respond to disease. This review examines recent progress using this technology across major livestock species. Scientists have created detailed cellular maps of healthy animals and discovered how specific cell types respond to important diseases like mastitis in dairy cows and viral infections in pigs. These findings are helping researchers design better vaccines, identify animals with stronger natural immunity for breeding programs, and develop new treatments. This work ultimately supports healthier animals, more sustainable farming, and safer food for consumers worldwide.

## 1. Introduction

Livestock species play a central role in global food security and agricultural sustainability, providing essential protein sources while supporting the livelihoods of millions worldwide [1]. The health and productivity of these animals are continuously challenged by infectious diseases, which impose substantial economic losses and threaten animal welfare. Effective control of these diseases relies on a detailed understanding of livestock immune systems to support the development of efficacious vaccines, targeted therapeutics, and evidence-based disease management strategies [2,3,4]. However, conventional bulk RNA sequencing approaches, while valuable for characterizing overall transcriptional responses, inherently obscure the cellular heterogeneity that defines functional immune architecture by collapsing diverse cell population signatures into averaged profiles [5]. This limitation has historically constrained our ability to identify specific immune cell subsets driving protective responses, to understand cell type-specific pathogen interactions, and to rationally design vaccines targeting defined cellular populations.

**Figure 1:**
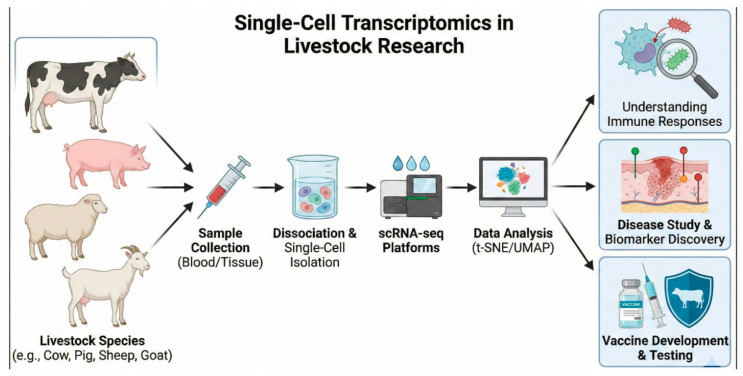
Single-cell RNA sequencing workflow and applications in livestock health research.

Single-cell RNA sequencing (scRNA-seq) has emerged as a transformative technology capable of resolving this complexity (Figure 1). By enabling transcriptomic characterization at individual cell resolution, scRNA-seq reveals dimensions of immune system organization and function previously inaccessible through population-level analyses [2,6,7,8,9]. Application of this technology to livestock species has accelerated dramatically since 2018, propelled by decreasing sequencing costs, refined tissue dissociation and cell capture protocols, and growing recognition of its analytical power for dissecting complex biological systems. Major collaborative initiatives—notably the Farm Animal Genotype-Tissue Expression (FarmGTEx) consortium—have generated comprehensive multi-tissue cellular atlases that now serve as foundational resources for the livestock research community. These efforts have systematically characterized cellular heterogeneity across dozens of tissues in cattle, pigs, and poultry while enabling comparative analyses with human and murine immunology that illuminate both evolutionarily conserved mechanisms and species-specific adaptations.

The deployment of scRNA-seq across production animal species has substantially advanced understanding of immune cell diversity and functional specialization. Pioneering applications in chickens [10] and pigs [11] established methodological frameworks subsequently extended to cattle [12,13,14,15]. Studies in cattle have revealed cell type-specific inflammatory responses, including preferential NF-κB pathway activation within monocyte and dendritic cell compartments following lipopolysaccharide stimulation [13], while comprehensive profiling of bovine mesenteric lymph nodes has delineated the heterogeneous landscape of mononuclear phagocyte populations and their distinct roles in antigen presentation and adaptive immune regulation [14]. Beyond conventional livestock, single-cell approaches have addressed longstanding limitations in immunological research on non-traditional agricultural species. In horses, where species-reactive immunological reagents remain scarce, scRNA-seq of peripheral blood mononuclear cells identified thirty distinct transcriptional clusters revealing conserved immune subsets analogous to human populations, including the unexpected predominance of T-bet-expressing B cells—a subset typically associated with chronic inflammation—establishing a foundational cellular atlas for equine immunology [16].

This review synthesizes advances in single-cell transcriptomics applied to livestock health, with an emphasis on immune system organization and disease responses. We focus on major production species, including cattle (*Bos taurus*), pigs (*Sus scrofa*), sheep (*Ovis aries*), and goats (*Capra hircus*), while incorporating insights from related agricultural species where relevant. We first examine the development of comprehensive single-cell atlases that provide reference frameworks for livestock genomics. We then discuss studies characterizing immune cell diversity and functional specialization, followed by investigations of host–pathogen interactions at single-cell resolution. Finally, we address current technical and analytical challenges and outline future directions, including the integration of spatial transcriptomics and multi-omics approaches, highlighting the implications of these advances for vaccine development, genetic improvement, and translational biomedical research.

## 2. Single-Cell Transcriptomic Atlases Across Major Livestock Species

The construction of comprehensive cell atlases represents a paradigm shift in livestock genomics, moving from tissue-level transcriptomics to systematic cataloguing of cellular diversity at single-cell resolution. Inspired by the Human Cell Atlas initiative, collaborative efforts across livestock species have generated foundational reference maps that serve three critical functions: establishing cell type taxonomies for standardized research, enabling integration of cellular phenotypes with genetic variation data, and facilitating cross-species comparisons that position agricultural animals as biomedical models. These atlases transform our capacity to understand the cellular basis of economically important traits and disease susceptibility.

### 2.1. Bovine Cell Atlases: Scale, Integration, and Translational Value

The Cattle Cell Atlas represents the most comprehensive single-cell resource for any livestock species, comprising nearly 1.8 million cells across 59 tissues with annotation of 131 distinct cell types [17]. This resource systematically characterizes intra-tissue and inter-tissue heterogeneity in gene expression, transcription factor regulation, and intercellular communication networks, establishing a reference framework for bovine biology. Critically, the atlas enables functional interpretation of genetic variants: integrative analysis with loci underlying monogenic and complex traits identified the specific cell types mediating genetic effects, including spermatocytes governing sperm motility and excitatory neurons influencing milk fat yield [18]. This cell type-to-trait mapping provides mechanistic resolution previously unattainable through bulk tissue approaches and opens new avenues for genomic selection strategies targeting specific cellular populations.

Beyond production trait genetics, bovine atlases have illuminated tissue-specific biology with direct agricultural applications. Skeletal muscle profiling identified fibro-adipogenic progenitor (FAP) subpopulations that regulate intramuscular adipogenesis and fibrogenesis—the cellular determinants of marbling—with comparative analysis across Wagyu and Brahman breeds revealing breed-specific regulatory mechanisms underlying meat quality variation [19]. Gastrointestinal characterization established the forestomach epithelium as the dominant site of antioxidant activity and oxidative phosphorylation [20], positioning this tissue as central to ruminant metabolic efficiency. Even specialized structures have been catalogued: intervertebral disc profiling identified 15 unique clusters annotated as nucleus pulposus, annulus fibrosus, notochord, muscle, endothelial, and immune cells, providing foundational data for understanding disc homeostasis, degeneration, and potential regenerative interventions [21].

A distinctive contribution of bovine atlases is their translational utility for human biomedical research. Comparative analysis revealed substantial gene expression similarities between cattle and humans, enabling identification of bovine cell types relevant for studying human complex phenotypes [17]. This positions cattle not merely as agricultural commodities but as valuable large-animal models where physiological scale, longevity, and outbred genetics more closely approximate human biology than traditional rodent models—a consideration increasingly relevant for preclinical research and xenotransplantation studies.

### 2.2. Porcine Atlases: Multi-Tissue Coverage and Biomedical Model Validation

Porcine single-cell resources have achieved comparable breadth to bovine atlases while emphasizing the pig’s dual role in agriculture and biomedical research. The PigGTEx Atlas, paralleling the human GTEx consortium, characterized 19 tissues with annotation of 67 major cell types [22]. This resource enables deconvolution of bulk RNA-seq samples to infer cellular composition, identification of cell-type-interaction expression quantitative trait loci (ieQTLs), and assignment of cellular mechanisms to 268 complex traits—creating direct links between genetic variation, cell type-specific gene regulation, and phenotypic outcomes. A complementary atlas of the Bama pig spanning seven tissues identified potential cell type marker genes, contributing to the growing infrastructure for precision porcine genomics [23].

Organ-specific atlases have revealed tissue biology with both agricultural and biomedical implications. Brain regional profiling across frontal, parietal, temporal, and occipital lobes plus hypothalamus identified 21 cell subpopulations, with cross-species comparison to mouse enabling analysis of pathways linked to neurological disorders [24]. This resource establishes the pig as a neurological disease model with greater translational relevance than rodents. Gastrointestinal profiling discovered novel intestinal lymphocyte populations bearing remarkable similarity to human immune cells, reinforcing the pig’s value for mucosal immunology research [11]. Kidney mapping across developmental periods and anatomical sites characterized 29 cell types with confirmed high transcriptional similarity to human counterparts [25]. Hepatic characterization [26] and gastrulation atlas construction Simpson et al. [27] further extend this comprehensive organ coverage.

Developmental and lifespan studies have leveraged the pig’s relatively long lifespan—compared to rodent models—to characterize temporal dynamics of cellular populations. Skin profiling across ten developmental stages from embryonic to seven years of age revealed cellular heterogeneity trajectories throughout development, aging, and across anatomical regions [28,29]. Immune cell dynamics proved particularly informative: dendritic cells (DCs) and T cells decreased perinatally while cytotoxic NKT cells peaked during rapid growth, with SPP1 and TGF-β signaling networks regulating cutaneous immunity across the lifespan. These temporal profiles provide essential baselines for understanding age-related immunosenescence and skin pathology. Reproductive biology has similarly benefited from breed-specific testicular atlases in Meihua and Hezuo pigs [30,31], contributing to understanding of male fertility determinants. The functional annotation of the pig transcriptome through network analysis established foundational genomic resources supporting these single-cell efforts [32].

### 2.3. Small Ruminant Atlases: Reproductive Biology and Breed-Specific Applications

Single-cell characterization in sheep and goats has followed a more targeted trajectory than the comprehensive multi-tissue efforts in cattle and pigs, with particular emphasis on reproductive biology and breed-specific economically important traits. This focused approach reflects both the agricultural priorities in small ruminant production and the more limited genomic resources historically available for these species. Foundational bulk transcriptomic atlases establishing baseline gene expression across tissues [33,34] provided essential reference data preceding single-cell applications.

Testicular development has received concentrated attention, driven by the economic importance of male fertility in breeding programs. In sheep, characterization of pre-sexual maturity stages [35] and dynamic transcriptional profiling of Hu sheep testes from birth through maturity [36] revealed spermatogenesis trajectories with identification of six somatic and five germ cell subtypes [37]. Goat studies have provided comparable resolution: dairy goat testis profiling identified six somatic and five spermatogenic cell subtypes with key regulatory roles for Notch, TGF-β, and Hippo signaling pathways, and established TKTL1 and AES as spermatogonial marker genes [38]. Developmental mapping across 45, 90, and 180-day-old animals characterized germ cell maturation alongside niche-related pathways including testosterone, retinoic acid, PDGF, FGF, and WNT signaling [39]. Female reproductive aging in goats has been addressed through single-cell atlas construction of the aging ovary [40], providing insight into fertility decline mechanisms.

Developmental biology studies have extended beyond reproduction. A single-cell atlas of early Mongolian sheep embryos at embryonic day 16 identified 13 and 8 major cell types in Ujumqin and Hulunbuir breeds, respectively, revealing signaling pathway activity—TGF-β, Hippo, and Wnt—in notochord, spinal cord, and paraxial mesoderm clusters associated with tail development [41]. This work provides cellular-level insight into breed-specific morphological variation with potential applications for understanding vertebral number determination.

Cashmere production represents a unique agricultural priority where single-cell approaches have proven particularly valuable. Hair follicle morphogenesis mapping [42] and elucidation of hair cycle regulation and apoptosis mechanisms [43] provide mechanistic understanding of fiber production with direct implications for genetic improvement programs targeting cashmere quality and yield. These studies exemplify how single-cell technologies can address species-specific agricultural traits not represented in model organism research.

Collectively, single-cell atlases across cattle, pigs, sheep, and goats have established cellular reference frameworks that fundamentally advance livestock genomics. Several common themes emerge from cross-species comparison. First, the integration of single-cell data with genetic variant information enables cell type-specific interpretation of GWAS signals and QTL effects, transforming abstract genetic associations into mechanistic hypotheses testable at cellular resolution. Second, cross-species transcriptomic similarity—particularly between pigs and humans in immune, neurological, and gastrointestinal systems—validates agricultural animals as biomedical models and suggests evolutionary conservation of cellular programs. Third, the predominant focus on reproductive biology in small ruminants compared to multi-tissue coverage in cattle and pigs reflects both agricultural priorities and resource availability, highlighting opportunities for expanded atlas construction in sheep and goats.

Critical gaps remain in current livestock cell atlases. Reference annotation quality for livestock species still lags behind human and mouse resources, complicating automated cell type identification. Temporal and environmental dynamics—how cellular populations shift with age, physiological state, and pathogen exposure—require longitudinal atlas construction beyond current snapshots. Integration of single-cell transcriptomics with spatial technologies, proteomics, and epigenomics will be essential for fully resolving the cellular basis of complex traits. Nevertheless, the foundational resources now available across major livestock species establish an infrastructure for precision animal breeding, disease resistance enhancement, and translational research applications that was inconceivable a decade ago. Table 1 provides a comprehensive summary of major single-cell atlases across livestock species, including tissue coverage, cell type annotations, and key findings with relevance to agricultural and biomedical applications.

## 3. Applications of Single-Cell Transcriptomics in Livestock Health

### 3.1. Immune Cell Characterization

**Figure 2:**
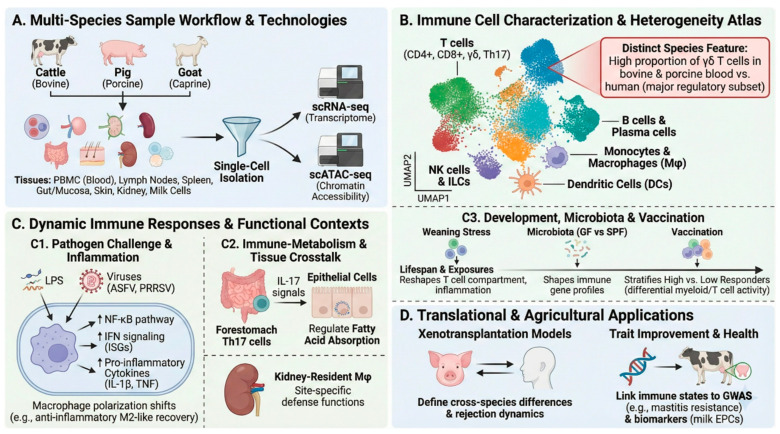
Single-cell transcriptomics elucidates immune cell heterogeneity and dynamic responses across livestock species. (**A**) Workflow showing diverse tissues sampled from cattle, pigs, and goats subjected to single-cell RNA sequencing (scRNA-seq) and integrated multi-omics (scATAC-seq) to profile immune cells. (**B**) Schematic UMAP visualization of major immune cell lineages characterized across species, including T cells, B cells, monocytes/macrophages, DCs, and natural killer (NK) cells. A prominent feature in bovine and porcine systems is the high proportion of γδ T cells compared to humans. (**C**) Functional characterization of immune responses in varied biological contexts. (**C1**) Response to pathogens viruses like African swine fever virus (ASFV), Porcine reproductive and respiratory syndrome virus (PRRSV) and bacterial LPS involves activation of NF-κB and interferon pathways, cytokine cascades, and dynamic shifts in macrophage polarization. (**C2**) Tissue-resident immune cells engage in metabolic crosstalk, such as bovine forestomach Th17 cells regulating fatty acid absorption and porcine kidney-resident macrophages demonstrating site-specific defense functions. (**C3**) Immune landscapes are reshaped by development (e.g., weaning stress in piglets), commensal microbiota colonization, and vaccination status (differentiating high vs. low responders). (**D**) Applications of single-cell atlases include informing xenotransplantation strategies through cross-species comparison and integrating immune data with GWAS to improve animal health traits and product quality surveillance (e.g., milk somatic cells).

The inherent heterogeneity of immune cell populations presents a fundamental challenge for understanding host defense mechanisms, as bulk sequencing approaches obscure the functional diversity within nominally homogeneous cell types [44]. Single-cell transcriptomics has emerged as the definitive tool for resolving this complexity, enabling precise characterization of immune cell subsets, their activation states, and intercellular communication networks across livestock species (Figure 2).

#### 3.1.1. Bovine Immune Architecture

Comprehensive single-cell profiling of bovine peripheral blood mononuclear cells (PBMCs) has established a foundational taxonomy of cattle immune populations. Integrated scRNA-seq and scATAC-seq analysis identified seven major immune cell types—CD4+ T cells, CD8+ T cells, B cells, monocytes, natural killer (NK) cells, innate lymphoid cells (ILCs), and DCs—while simultaneously mapping chromatin accessibility landscapes that govern lineage-specific gene expression [13]. This multi-omic framework revealed that lipopolysaccharide (LPS) stimulation dynamically remodels both transcriptional programs and chromatin architecture, with the NF-κB pathway serving as a central hub coordinating innate immune activation and pro-inflammatory cytokine production. Critically, time-course analysis across 0, 2, 4, and 8 h post-LPS stimulation captured the kinetics of immune activation, and correlation with genome-wide association data linked specific immune cell states to complex traits including mastitis susceptibility [45].

Beyond peripheral blood, tissue-resident immune populations exhibit striking functional specialization. Mononuclear phagocyte profiling from bovine mesenteric lymph nodes revealed seventeen distinct clusters—ten dendritic cell and seven monocyte/macrophage populations—each with unique transcriptomic signatures [14]. Among DCs, the study distinguished lymph node-resident subsets from highly activated migratory populations characterized by elevated expression of T cell-attracting chemokines. Monocyte and macrophage subclustering reflected functional polarization along pro- and anti-inflammatory axes, including identification of cycling macrophages suggestive of local self-renewal capacity. This heterogeneity underscores that immune responses cannot be adequately characterized through peripheral blood sampling alone.

A distinctive feature of bovine immunity is the prominence of γδ T cells, which constitute a substantially higher proportion of peripheral blood lymphocytes compared to humans, where αβ T cells predominate [46,47]. Single-cell analysis of vaccine responses in Angus cattle stratified by delayed-type hypersensitivity (DTH) responses demonstrated that this cell population contributes differentially to protective immunity: high-responder animals exhibited elevated CD8^−^ γδ T cell activity alongside pro-inflammatory myeloid signatures, whereas low responders showed predominant NKT cell inflammatory responses [2]. Intercellular communication analysis identified the IL-1β–IL-1R1 axis as a critical determinant of vaccine efficacy [48], suggesting that modulation of innate inflammatory cascades may enhance vaccination outcomes.

The intersection of immunity and metabolism represents an emerging frontier with particular relevance to ruminant biology. A single-cell atlas of ten metabolic tissues from lactating dairy cattle identified tissue-specific immune cell enrichment patterns, most notably the localization of T helper 17 (Th17) cells within forestomach tissues [49]. These Th17 cells engage epithelial populations through IL-17 signaling to regulate transcriptional programs governing short-chain fatty acid absorption—a finding that reveals how immune cells directly modulate metabolic functions essential for milk production and positions immunity as an integral component of productive efficiency rather than merely a defensive system.

#### 3.1.2. Porcine Immune Diversity and Cross-Species Conservation

The pig represents both a major agricultural species and an increasingly important biomedical model, making comprehensive immune characterization essential for veterinary and translational applications. Foundational atlases profiling porcine PBMCs through integrated bulk and single-cell RNA sequencing resolved 13 general cell types across nearly 29,000 cells, with cross-species comparison revealing conserved immune architecture between pigs and humans [50]. However, direct scRNA-seq comparison of human and porcine PBMCs identified important distinctions: pigs harbor fewer T cells, NK cells, and monocytes but greater B cell proportions, with heightened metabolic pathway activity in porcine monocytes and reduced cytotoxicity signatures in CD8+ T cells [51]. These differences have direct implications for xenotransplantation, where understanding species-specific immune features is essential for engineering immunologically compatible donor animals.

Tissue-specific immune profiling has revealed remarkable functional specialization across organ systems. A macrophage atlas derived from 49 tissues of pregnant pigs identified 33 distinct subtypes with extensive tissue-specific diversity [52]. The Mφ MARCO+ subtype in mesenteric adipose tissue exhibited heightened pattern recognition receptor signaling compared to other anatomical sites, while lung macrophages showed unique expression of PLSCR1. Similarly, kidney-resident macrophages demonstrated site-specific functional divergence: medullary populations specialized in phagocytosis and leukocyte activation, whereas pelvic macrophages recruited bactericidal neutrophils [25]. Cross-species analysis confirmed high transcriptional similarity between porcine and human kidney cell types, further validating the pig as a translational model.

Developmental and environmental factors profoundly shape porcine immune landscapes. Single-cell profiling of pig skin across the lifespan revealed dynamic immune cell trajectories: DCs and T cells decrease perinatally, while cytotoxic NKT cells peak during rapid growth phases [53]. These populations utilize SPP1 and TGF-β signaling networks to regulate cutaneous immunity during development and aging, with cross-species analysis confirming evolutionary conservation of these functional profiles with humans. The pig thymus atlas further elucidated T cell development, identifying unconventional T cell types with innate effector profiles and characterizing over 11,000 differentiating invariant natural killer T (iNKT) cells—revealing that porcine iNKT functional diversity diverges significantly from the established mouse paradigm [54].

The microbiome exerts substantial influence over immune cell identity and function. Comparative profiling of immune tissues from germ-free and specific pathogen-free piglets across 57,720 cells demonstrated that commensal microbiota colonization significantly alters gene expression profiles of B cells, T cell subsets, and myeloid populations across Peyer’s patches, mesenteric lymph nodes, and spleen [53]. Weaning stress similarly reshapes the immune compartment: ileal mucosa profiling from suckling and weaned piglets revealed T cell compartment remodeling, with Th17 plasticity and granzyme B-expressing cytotoxic T cell enrichment driving mucosal inflammation through epithelial mitochondrial dysfunction [55]. These findings establish early-life environmental exposures as critical determinants of immune programming.

Domestication itself has imposed selective pressures on immune function. Comparative scRNA-seq of jejunal tissues from wild boars and domesticated breeds (Jinhua, Duroc) across over 26,000 cells demonstrated largely conserved immune cell functions but stronger overall immune capabilities in wild populations [56]. Domestication drove breed-specific gene expression patterns linked to metabolism and immune surveillance, including identification of a unique plasma cell population with distinct antibody production in Jinhua pigs. These findings provide resources for breeding programs aimed at enhancing immune traits while maintaining productive efficiency.

#### 3.1.3. Small Ruminant Immune Characterization

Single-cell characterization of small ruminant immunity remains less developed than for cattle and pigs, though recent studies have begun addressing this gap. The first single-cell atlas of somatic cells in goat milk identified seven distinct populations and revealed substantial inter-individual variability in epithelial cell proportions correlating with somatic cell count [57]. This variability defined two immunologically divergent states: high epithelial cell samples exhibited pro-inflammatory signaling with enriched SAA and PAEP expression, while low epithelial cell samples showed anti-inflammatory markers including SERPIN B3 and C3 alongside TGF-β/SPP1 signaling. This work positions epithelial cell proportion as a cellular biomarker linking mammary immune status to milk quality—a finding with direct implications for dairy production management and food safety surveillance.

### 3.2. Single-Cell Transcriptomics in Disease Response Studies

Beyond baseline characterization, single-cell transcriptomics has proven transformative for understanding host-pathogen interactions, revealing infection dynamics, cellular tropism, and immune evasion mechanisms inaccessible through conventional approaches. These studies not only advance fundamental understanding of disease pathogenesis but also identify molecular targets for therapeutic intervention and rational vaccine design.

#### 3.2.1. Bovine Mastitis: From Cellular Mechanisms to Therapeutic Targets

**Figure 3:**
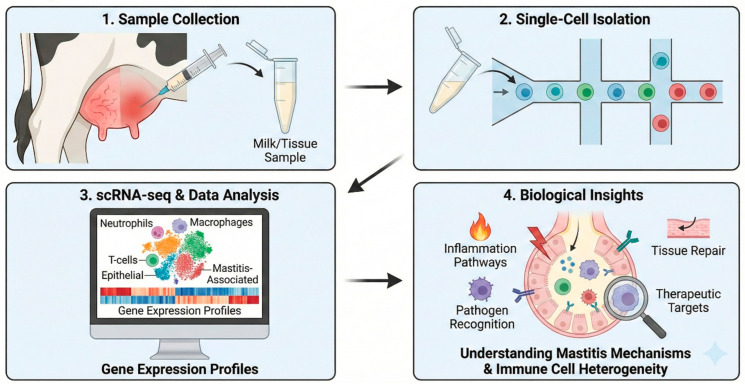
Schematic overview of single-cell RNA sequencing (scRNA-seq) workflow for investigating bovine mastitis.

Mastitis remains the most economically significant disease affecting the global dairy industry, and single-cell technologies have fundamentally advanced mechanistic understanding of mammary inflammation (Figure 3). Foundational work established reference frameworks through Drop-seq profiling of bovine milk cells and primary mammary epithelial cells, revealing substantial intrapopulation heterogeneity [58]. Building upon this foundation, characterization of chronic *Staphylococcus aureus* infection in Holstein cattle identified milk-enriched granulocyte subpopulations with transcriptional signatures governing chemotaxis, myeloid differentiation, and inflammatory cascades—demonstrating that the local mammary microenvironment shapes immune cell functional states in ways not reflected in peripheral blood [12].

The intersection of immune function and lactation performance has emerged as particularly productive. Differential pathway activation between high- and low-producing animals revealed enhanced prolactin signaling and distinct Toll-like receptor and NF-κB regulatory patterns in high-yield phenotypes, alongside epithelial-immune communication axes mediated by cyclophilin A, ICAM, and SELL signaling [7]. Integration of immune-responsive gene modules with genome-wide association data has linked specific cellular states to mastitis susceptibility and milk production traits [13], establishing a framework for genomic selection strategies targeting disease resistance. Therapeutic translation remains challenging: while milk-derived stem/progenitor cells possess secretomes capable of inhibiting mastitis-associated pathogens including antibiotic-resistant strains [59], randomized trials demonstrated superior bacteriological cure rates with conventional antibiotic therapy compared to platelet-rich plasma despite distinct cytokine kinetics [60]—underscoring the continued need to bridge cellular insights with practical treatment strategies.

#### 3.2.2. Bacterial Infections in Small Ruminants

*Mycoplasma ovipneumoniae*, a causative agent of chronic respiratory disease in sheep, exemplifies how single-cell approaches illuminate bacterial pathogenesis. Lung tissue profiling at 28 days post-infection identified 11 distinct cell populations and demonstrated substantially inhibited intercellular signaling during chronic infection, with cyclophilin A and macrophage migration inhibitory factor pathways playing critical regulatory roles [61]. The infection decreased CD8+ effector T cell cytotoxicity while depleting regulatory T cells, and S100A9 emerged as a neutrophil marker promoting bacterial clearance through ERK signaling and reactive oxygen species production. Similarly, *Brucella* infection in goat testicular tissues revealed dynamic immunological remodeling with T cell hyperactivation linked to CD45-mediated signaling, identification of thioredoxin-interacting protein as a potential immunotherapeutic target, and disrupted CD39- and JAM-dependent intercellular communication contributing to erosion of testicular immune privilege [52]. These studies demonstrate the power of single-cell resolution for identifying candidate therapeutic targets in livestock bacterial diseases.

#### 3.2.3. Swine Viral Pathogens: Tropism, Evasion, and Conserved Defense Mechanisms

African swine fever virus. ASFV represents one of the most devastating threats to global swine production, and single-cell transcriptomics has provided unprecedented resolution of infection dynamics. The first comprehensive scRNA-seq analysis of ASFV-infected primary porcine alveolar macrophages (PAMs) revealed increased viral transmembrane gene expression and identified TNF-α production as necessary for ASFV-induced apoptosis [62]. The unfolded protein response pathway showed differential regulation—activated in cells with low viral loads but suppressed in highly infected cells—suggesting viral manipulation of host stress responses. Comparative analysis demonstrated that attenuated and low-virulence strains paradoxically exhibit higher viral loads due to upregulated RNA polymerase subunit expression, with an IRF7-mediated positive feedback loop driving interferon signaling in cells exposed to these strains [63]. Two PAM subpopulations marked by CD163 and SIGLEC1 expression produce high levels of interferon-stimulated genes and IL-18, respectively, representing functionally distinct antiviral responders.

Splenic infection studies identified macrophages and monocytes as major infected cell types but revealed a critical finding: rare CD14-negative immature monocytes become the predominant infected population at late infection stages, exhibiting inhibited apoptosis, dampened interferon responses, and reduced antigen presentation that facilitate prolonged infection in vivo [64]. Further characterization identified four functionally distinct macrophage subsets, including an ‘AntiviralMac’ population rapidly depleted during infection and a metabolically reprogrammed ‘SusceptibleMac’ population serving as the major viral niche, with E165R identified as a central hub in viral replication networks and disruption of Netrin signaling pathways potentially facilitating immune evasion [65].

Porcine reproductive and respiratory syndrome virus. PRRSV infection dynamics reveal complex immunological responses characterized by aberrant immune cell differentiation. Virulence determines disease trajectory: highly virulent strains cause faster replication with earlier peak lung damage, while less than 5% of macrophages are directly infected—implicating bystander cell death potentially mediated by exosomal microRNAs [66]. SPP1+ macrophages serve as primary target cells, with extensive apoptosis causing significant macrophage reduction and compensatory monocyte differentiation [67]. The identification of SLAMF7 as a key gene in highly infected subpopulations, with knockdown significantly reducing viral replication, suggests potential therapeutic targets [68]. Cells with highest viral expression interact with neighboring populations via the SPP1-CD44 complex, and exogenous SPP1 protein demonstrates antiviral potential [69]. Anti-inflammatory M2-like macrophages (SPP1-CXCL14 positive) increase during the recovery phase of intermediate-virulence infections, contributing to lung repair—a response notably absent in high-virulence infection [66].

Other swine viral pathogens. Porcine circovirus type 2 (PCV2) pathogenesis involves disruption of B cell differentiation, with decelerated differentiation of light zone germinal center B cells into memory B cells and plasma cells potentially explaining impaired vaccine efficacy [70]. Porcine epidemic diarrhea virus (PEDV) preferentially infects enterocytes, goblet cells, and tuft cells—the latter marked by a novel marker, DNAH11 [71]. Despite intestinal damage, enhanced epithelial repair occurs through increased stem cell proliferation and differentiation into enterocytes, with IFN-γ and IL-10 signaling serving as critical regulators of immune balance and tissue homeostasis [65].

Conserved antiviral mechanisms. Comparative analysis across ASFV and PRRSV infections revealed conserved defense pathways, identifying pattern recognition receptors RIG-I, MDA5, and LGP2 as key sensors recognizing both viruses [72]. Both infections trigger upregulation of CCL, CXCL, IL, and TNF cytokine families alongside pyroptosis induction. Weighted gene co-expression network analysis identified six proteins—PARP12, PARP14, HERC5, DDX60, RSAD2, and MNDA—as potential inhibitors of both pathogens, suggesting shared therapeutic targets applicable across swine viral diseases.

#### 3.2.4. Translational Applications: Xenotransplantation and Vaccine Development

Single-cell characterization of porcine immunity has direct translational implications. Analysis of a human recipient of a pig liver xenograft revealed key immune dynamics driving rejection: progressive T cell activation in peripheral blood, infiltration of the graft by γδT and exhausted T cells, and identification of two critical monocyte subsets—early THBS1+ monocytes regulating coagulation via platelet interaction, and later-infiltrating C1QC+ monocytes promoting T cell exhaustion through PD-L1 induction [57]. These findings position innate immune cells as critical arbiters of thrombotic and adaptive immune pathways in xenotransplantation.

For vaccine development, understanding the B cell repertoire is essential. A high-throughput single-cell method simultaneously profiling global and immunoglobulin transcriptomes of up to 10,000 individual pig B cells enables comprehensive repertoire mapping and recovery of paired antibody sequences [73]. Similarly, simultaneous single-cell transcriptome and immune receptor profiling in pigs re-exposed to influenza A virus revealed expanded T cell clonotypes with activated phenotypes, suggesting identification of antigen-reactive clones [54]. These methodological advances will directly inform development of improved swine and human vaccines while enhancing preparedness against emerging zoonotic threats. Table 2 summarizes key single-cell transcriptomic studies of disease responses across livestock species, detailing methods, disease models, and principal findings.

### 3.3. Technical Considerations and Methodological Challenges

The transformative impact of scRNA-seq on livestock immunology must be contextualized within an understanding of inherent technical limitations that shape data interpretation. Platform selection represents a fundamental trade-off between breadth and depth of transcriptomic coverage. Droplet-based systems—exemplified by the widely adopted 10x Genomics Chromium platform—enable high-throughput profiling of tens of thousands of cells per sample, yet capture only 2000–5000 unique transcripts per cell on average due to low capture efficiency (~10–20%) [77]. This limited sensitivity may fail to detect lowly expressed transcription factors, cytokines, and regulatory molecules that govern immune cell fate decisions and functional responses. Conversely, plate-based full-length transcript approaches (e.g., Smart-seq2, Smart-seq3) achieve near-complete transcript coverage with detection of 10,000–15,000 genes per cell, enabling splice variant analysis and improved detection of key immune regulators, but at the cost of substantially reduced throughput (hundreds to low thousands of cells) and higher per-cell cost [78]. This constraint limits statistical power for identifying rare cell populations and restricts the scope of comparative analyses across multiple conditions or individuals.

Tissue processing introduces systematic biases with cell type-specific consequences. Enzymatic dissociation protocols—typically employing collagenase, dispase, or other proteolytic enzymes—preferentially lyse fragile cell types while resilient populations survive, artifactually skewing apparent cell frequencies. Neutrophils and activated lymphocytes may be disproportionately lost during processing, while stromal cells and tissue-resident macrophages are overrepresented. The mechanical and enzymatic stress of dissociation can induce artifactual gene expression changes, including immediate-early stress response genes (e.g., FOS, JUN, EGR1) that confound interpretation of activation states [79]. Comparisons between tissue-resident and circulating immune populations are particularly vulnerable to these artifacts, as circulating cells experience minimal processing while tissue-derived cells undergo extensive manipulation. Single-nucleus RNA sequencing offers an alternative that bypasses some dissociation artifacts and enables profiling of frozen archived samples, but sacrifices cytoplasmic transcripts including many immune effector molecules [80].

Computational and annotation challenges remain substantial obstacles in livestock species. Genome assemblies for cattle, pigs, sheep, and goats continue to lag behind human and mouse resources in both contiguity and annotation completeness [81]. Incomplete gene models lead to unmapped reads, while absent or misannotated genes—including key immune markers—complicate cell type identification and cross-study comparisons. Automated cell type annotation tools trained predominantly on human and mouse atlases may fail to recognize livestock-specific immune populations or project inappropriate cell type labels onto genuinely distinct cellular states [82]. The numerical dominance of γδ T cells in ruminant blood—a minor population in humans and mice—exemplifies this challenge. Cross-species integration analyses, while valuable for comparative immunology, risk imposing human/mouse-centric frameworks onto livestock immune systems with distinct evolutionary adaptations. Batch effects arising from differences in tissue handling, sequencing platforms, library preparation kits, and computational pipelines further complicate meta-analyses and data integration efforts. Addressing these limitations requires continued investment in genome annotation, development of livestock-specific reference atlases, and implementation of robust batch correction and integration methods.

The livestock single-cell literature contains apparent contradictions that warrant careful interpretation. Studies of bovine mastitis report varying cellular compositions in infected milk, with some identifying neutrophils as the predominant immune infiltrate while others report macrophage dominance. These divergent findings likely reflect genuine biological heterogeneity rather than technical artifacts. Infection stage represents a critical variable: early acute mastitis is characterized by massive neutrophil recruitment, while chronic infections transition to macrophage-dominated responses. Pathogen identity further modulates cellular composition—Gram-positive organisms (e.g., *Staphylococcus aureus*, *Streptococcus* species) and Gram-negative bacteria (e.g., *Escherichia coli*) elicit distinct immune responses with different kinetics and cellular players [83]. Individual animal variation in immune responsiveness, influenced by genetics, parity, lactation stage, and concurrent health status, contributes additional heterogeneity. Sampling methodology—including timing of sample collection relative to infection onset, milk fraction analyzed (foremilk vs. composite), and cell isolation protocols—introduces further variation.

Similarly, PRRSV tropism studies vary in reported macrophage subset preferences and infection dynamics. While SPP1+ alveolar macrophages consistently emerge as primary target cells across studies, the proportion of infected cells, viral load distributions, and bystander cell responses differ between reports. These variations reflect strain-specific differences in viral tropism and virulence, as well as methodological differences in macrophage subpopulation definitions. The lack of standardized macrophage nomenclature across livestock species—comparable to the human macrophage ontology efforts—complicates direct cross-study comparisons. Host genetic background and prior immune experience also shape infection outcomes, with breed-specific differences in viral susceptibility documented in pigs [84].

Cross-species comparative studies occasionally report conflicting conclusions regarding conservation of immune cell types between livestock and humans. Some analyses emphasize remarkable transcriptomic similarity of orthologous cell populations, validating pigs and cattle as translational models. Others highlight substantial differences in cell frequencies, functional markers, and immune pathway utilization. These apparent contradictions reflect genuine species-specific immune adaptations rather than inconsistent findings. The high proportion of γδ T cells in ruminant blood, the expanded innate lymphoid cell compartments in porcine gut, and species-specific expression patterns of pattern recognition receptors represent legitimate biological differences shaped by evolutionary pressures including pathogen exposure, commensal microbiota composition, and metabolic physiology [84].

Resolution of these apparent discrepancies requires multi-pronged approaches. Larger sample sizes across diverse breeds, ages, infection stages, and environmental contexts will better capture the true biological variation underlying phenotypic heterogeneity. Standardized protocols for tissue processing, cell isolation, and computational analysis—analogous to initiatives in human immunology [82]—would reduce technical variation and improve reproducibility. Development of consensus cell type definitions and molecular markers specific to livestock species, informed by functional validation rather than solely transcriptomic similarity to human/mouse counterparts, will enable more accurate cross-study comparisons. Ultimately, the field must embrace biological heterogeneity as an inherent feature of livestock populations rather than experimental noise, recognizing that outbred animals raised in diverse production environments exhibit genuine immunological variation that both complicates interpretation and represents the reality of agricultural systems.

## 4. Conclusions and Future Perspectives

Single-cell RNA sequencing has transformed livestock immunology by revealing cellular heterogeneity and functional specialization beyond the reach of bulk transcriptomics. The development of single-cell atlases for major livestock species has provided essential references for studying immune organization, host–pathogen interactions, and tissue-specific immunity, enabling the identification of novel immune cell populations and species-specific features relevant to animal health and productivity. However, several challenges remain. Incomplete genome annotations, technical limitations in tissue processing, and batch effects hinder accurate cell identification and data integration, particularly in non-model species. In addition, robust frameworks linking single-cell phenotypes to complex disease and production traits are still under development. Future advances will rely on integrating emerging approaches, including spatial transcriptomics, multi-omics, and longitudinal single-cell studies, to better capture immune dynamics, regulation, and environmental responses. From an applied standpoint, single-cell technologies hold strong potential for improving vaccine design, disease diagnostics, and genetic selection for immune traits. As costs decrease and analytical tools mature, single-cell approaches are expected to become integral to livestock research, driving improvements in animal health, sustainability, and food security.

## Figures and Tables

**Table 1:** Comprehensive tissue atlases in livestock.

Species	Atlas Name	Tissues/Cell Types	References
Cattle	Cattle Cell Atlas (CattleCA)	59 tissues; 131 cell types/Linked specific cell types to economically important traits, serving as a foundational resource for bovine genetics, selective breeding, and comparative human biology.	[17]
	Bovine Skeletal Muscle Atlas	Skeletal muscle; 21 clusters, including fibro/adipogenic progenitor (FAP) subpopulations	[19]
	Bovine Redox-Metabolism Atlas	59 tissues; analysis focused on oxygen signaling states in 1006 clusters, particularly gastrointestinal tract epithelium.	[20]
	Bovine Intervertebral Disc Atlas	Caudal intervertebral disc (IVD); 15 unique clusters annotated as nucleus pulposus (NP), annulus fibrosus (AF), notochord, muscle, endothelial, and immune cells. To understand IVD development, homeostasis, degeneration, and regeneration	[21]
Pig	Chenghua Pig Skin Lifespan Atlas	Skin (10 developmental stages, embryonic to 7 years); identified 8 major cell types. Immune findings DCs and T cells decreased perinatally; cytotoxic NKT cells peaked at rapid growth stage. DCs/TCs utilized SPP1 and TGF-β signaling to regulate skin immunity.	[25]
	PigGTEx Atlas	19 tissues; annotated to 67 major cell types. Used to deconvolute bulk RNA-seq samples, identify cell-type-interaction eQTLs (ieQTLs), and provide cellular mechanisms for 268 complex traits.	[22]
	Pig Brain Regional Atlas	Brain (frontal, parietal, temporal, occipital lobes, hypothalamus); identified 21 cell subpopulations. Includes cross-species comparison with mouse and analysis of pathways linked to neurological disorders	[26]
Sheep	Mongolian Sheep Embryonic Atlas (E16)	Early embryo (E16); identified 13 (Ujumqin) and 8 (Hulunbuir) major cell types. Revealed signaling pathways (e.g., TGF-β, Hippo, Wnt) in notochord, spinal cord, and paraxial mesoderm clusters associated with tail development.	[41]
	Hu Sheep Testis Single-Cell Atlas	Testis; identified six somatic cell subtypes and five germ cell subtypes, revealing spermatogenesis trajectory.	[37]
Goat	Dairy Goat Testis Single-Cell Atlas	Testis; identified six somatic cell and five spermatogenic cell subtypes. Key pathways include Notch, TGF-β, and Hippo signaling. Marker genes TKTL1 and AES identified for spermatogonia.	[38]
	Dairy Goat Testis Developmental Atlas	Testis (45, 90, 180-day-old); cell populations include spermatogonia, spermatocytes, spermatids, Sertoli, Leydig, macrophages, endothelial cells. Mapped germ cell development and niche-related pathways (testosterone, retinoic acid, PDGF, FGF, WNT).	[39]

**Table 2:** Summary of Single transcriptomic application in livestock diseases.

Method	Animal Species	Disease/Challenge	Key Outcomes	References
scRNA-seq and flow cytometry	Pig (Swine)	H1N1pdm09 infection; Respiratory immunization with adenoviral vector vaccine (±IL-1β)	1. IL-1β adjuvant reduced functionally active Treg cells. 2. Influenza infection upregulated IFI6 in BAL cells, reducing susceptibility to virus replication in vitro. 3. Created a reference map of porcine BAL immune cells, distinguishing tissue-resident from circulating populations.	[74]
scRNA-seq		ASFV infection	1. The spleen is the most severely affected organ with the highest viral load. 2. Macrophages and monocytes are the major infected cell types, with high viral-load heterogeneity. 3. ASFV infection shifts from macrophages to a rare subpopulation of CD14-negative immature monocytes at late stages. 4. These immature monocytes have inhibited apoptosis, interferon response, and antigen presentation, facilitating prolonged ASFV infection in vivo.	[64]
		Porcine reproductive and respiratory syndrome virus (PRRSV) infection with strains of varying virulence	1. High virulence: Faster viral replication leads to earlier, severe lung damage with significant macrophage decreases and lymphocyte influx. <5% of macrophages are directly infected, implicating bystander cell death (potentially via exosomal miRNAs). 2. Intermediate virulence: Delayed peak lung damage with fewer cellular changes. 3. Key immune finding: SPP1-CXCL14high anti-inflammatory M2-like macrophages increase during peak damage in intermediate infection, aiding defense and recovery—a response absent in high-virulence infection.	[66]
		Influenza A virus (IAV) infection	1. All pigs presented highly diverse immune repertoires. 2. Pigs re-exposed to IAV showed more expanded T cell clonotypes with activated phenotypes, suggesting IAV-reactive clones. 3. Validated a high-throughput method for simultaneous single-cell transcriptome and immune receptor profiling in pigs.	[54]
	Pig (porcine alveolar macrophages)	ASFV infection	1. Antiviral and inflammatory pathways were activated, with increased interferon-stimulated and cytokine-related genes. 2. The unfolded protein response (UPR) was activated in low viral load cells but suppressed in high viral load cells. 3. ASFV promoted host metabolic pathways while inhibiting interferon and UPR signaling. 4. ASFV infection activated cell apoptosis, mediated by TNF-α production	[62]
	Pig (Primary porcine alveolar macrophages—PAMs)	ASFV infection	1. Attenuated/low-virulence strains showed higher viral loads, linked to upregulated viral RNA polymerase genes. 2. An IRF7-mediated positive feedback loop enhanced interferon signaling in cells exposed to attenuated/low-virulence strains. 3. Identified two key PAM subpopulations: IFI16+ and CD163+ cells, which produced high levels of interferon-stimulated genes (ISGs) and IL18 to regulate the host response.	[63]
	Pig (Piglets)	Porcine epidemic diarrhea virus (PEDV) infection	1. Enhanced epithelial repair via increased proliferation and differentiation of stem/TA/progenitor cells into enterocytes. 2. Disrupted intercellular communication and activated immune responses, with IFN-γ and IL-10 signaling as critical regulators. 3. PEDV initiated replication in B and T lymphocytes but failed to produce infectious progeny; T cells underwent virus-induced apoptosis.	[65]
	Pig (Piglets)	Porcine reproductive and respiratory syndrome virus (PRRSV) infection	1. Extensive apoptosis of macrophages led to a significant reduction in their numbers. 2. SPP1high macrophage subpopulation identified as the primary target cell for PRRSV infection. 3. Infection enhanced ligand-receptor interactions between macrophages and other cells, driving inflammation and immune cell activation. 4. Monocytes showed a tendency to differentiate into macrophages, possibly compensating for macrophage depletion. 5. Caused abnormal B cell development and incomplete activation of cytotoxic T lymphocytes in the lungs.	[67]
	Transgenic (metabolic disease-susceptible) and wild-type pigs	Metabolic disorder induced by a high-fat high-sucrose diet	1. Reparative LYVE1+ macrophages were lost in hearts with metabolic disorder. 2. Proinflammatory endothelial cells were activated, showing high expression of multiple cytokines. 3. Metabolically active cardiomyocytes exhibited impaired function and reduced abundance.	[75]
scRNA	Boer goats	Infection with Haemonchus contortus parasite	1. Identified seven immune cell types (T cells, monocytes, NK cells, B cells, DCs).	[76]

## Data Availability

No new data were created or analyzed in this study.
